# Canagliflozin reduces inflammation and fibrosis biomarkers: a potential mechanism of action for beneficial effects of SGLT2 inhibitors in diabetic kidney disease

**DOI:** 10.1007/s00125-019-4859-4

**Published:** 2019-04-17

**Authors:** Hiddo J. L. Heerspink, Paul Perco, Skander Mulder, Johannes Leierer, Michael K. Hansen, Andreas Heinzel, Gert Mayer

**Affiliations:** 1 Department Clinical Pharmacy and Pharmacology, University of Groningen, University Medical Center Groningen, Hanzeplein 1, PO Box 30 000, 9700 AD Groningen, the Netherlands; 2 Department of Internal Medicine IV (Nephrology and Hypertension), Medical University of Innsbruck, Innsbruck, Austria; 3 Cardiovascular and Metabolic Disease Research, Janssen Research & Development, LLC, Spring House, PA USA; 4 emergentec biodevelopment GmbH, Vienna, Austria

**Keywords:** Biomarkers, Canagliflozin, Chronic kidney disease, Sodium–glucose cotransporter 2 inhibitors, Type 2 diabetes

## Abstract

**Aims/hypothesis:**

The sodium–glucose cotransporter 2 (SGLT2) inhibitor canagliflozin slows progression of kidney function decline in type 2 diabetes. The aim of this study was to assess the effect of the SGLT2 inhibitor canagliflozin on biomarkers for progression of diabetic kidney disease (DKD).

**Methods:**

A canagliflozin mechanism of action (MoA) network model was constructed based on an in vitro transcriptomics experiment in human proximal tubular cells and molecular features linked to SGLT2 inhibitors from scientific literature. This model was mapped onto an established DKD network model that describes molecular processes associated with DKD. Overlapping areas in both networks were subsequently used to select candidate biomarkers that change with canagliflozin therapy. These biomarkers were measured in 296 stored plasma samples from a previously reported 2 year clinical trial comparing canagliflozin with glimepiride.

**Results:**

Forty-four proteins present in the canagliflozin MoA molecular model overlapped with proteins in the DKD network model. These proteins were considered candidates for monitoring impact of canagliflozin on DKD pathophysiology. For ten of these proteins, scientific evidence was available suggesting that they are involved in DKD progression. Of these, compared with glimepiride, canagliflozin 300 mg/day decreased plasma levels of TNF receptor 1 (TNFR1; 9.2%; *p* < 0.001), IL-6 (26.6%; *p* = 0.010), matrix metalloproteinase 7 (MMP7; 24.9%; *p* = 0.011) and fibronectin 1 (FN1; 14.9%; *p* = 0.055) during 2 years of follow-up.

**Conclusions/interpretation:**

The observed reduction in TNFR1, IL-6, MMP7 and FN1 suggests that canagliflozin contributes to reversing molecular processes related to inflammation, extracellular matrix turnover and fibrosis.

Trial registration ClinicalTrials.gov NCT00968812

**Electronic supplementary material:**

The online version of this article (10.1007/s00125-019-4859-4) contains peer-reviewed but unedited supplementary material, which is available to authorised users.

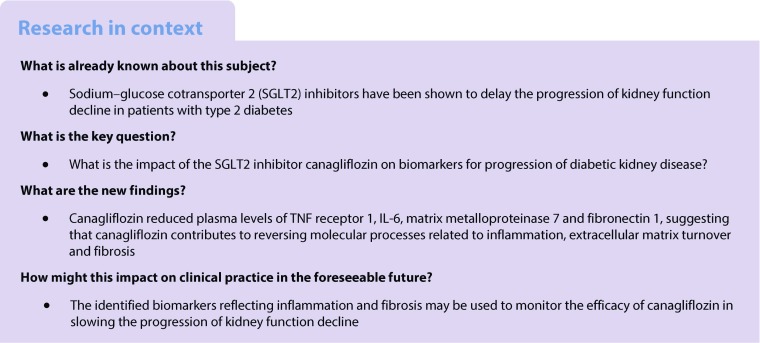



## Introduction

Chronic kidney disease (CKD) is present in 35–40% of individuals with type 2 diabetes. These individuals are at markedly increased risk of end-stage kidney disease, cardiovascular disease and premature mortality compared with individuals without CKD despite optimal recommended treatment [1]. Novel treatments are therefore highly desired to mitigate the high residual risk [2].

Sodium–glucose cotransporter 2 (SGLT2) inhibitors are a relatively new class of oral glucose-lowering drugs. They inhibit the SGLT2 transporter in the S1 segment of the proximal tubule in the kidney and cause glycosuria and natriuresis. Previous studies have shown that SGLT2 inhibitors improve several cardiovascular and renal risk factors including HbA_1c_, BP, body weight and albuminuria [3, 4]. Results from large cardiovascular outcome trials demonstrated that these beneficial effects translate into cardiovascular protection and delay of kidney function decline [5, 6]. The exact mechanisms by which SGLT2 inhibitors reduce the risk of cardiovascular and kidney disease are not completely understood but are thought to involve natriuresis, restoration of tubuloglomerular feedback and amelioration of intrarenal hypoxia [7]. In addition, experimental studies have suggested possible anti-inflammatory and antifibrotic effects for SGLT2 inhibitors [8, 9].

Advancements in omics technologies coupled with high-dimensional data integration via systems medicine approaches can provide new insights into the molecular mechanism of action (MoA) of drugs and pathways of disease progression [10]. In a recent study, we used publicly available and experimental data to develop a molecular process model of diabetic kidney disease (DKD) [11]. The model was subsequently used to derive and validate novel biomarkers predicting progression of DKD. The current study expands this work and uses a similar bioinformatics approach to construct a molecular MoA network model describing the impact of the SGLT2 inhibitor canagliflozin at a molecular level. Biomarker candidates for monitoring the efficacy of canagliflozin in slowing the progression of CKD in individuals with type 2 diabetes were selected based on network interference of the canagliflozin MoA and DKD pathophysiological process model. The candidate biomarkers were then validated in plasma samples from the Canagliflozin Treatment and Trial Analysis-Sulfonylurea (CANTATA-SU) trial, a phase 3 clinical trial comparing canagliflozin with glimepiride [12].

## Methods

**Fig. 1:**
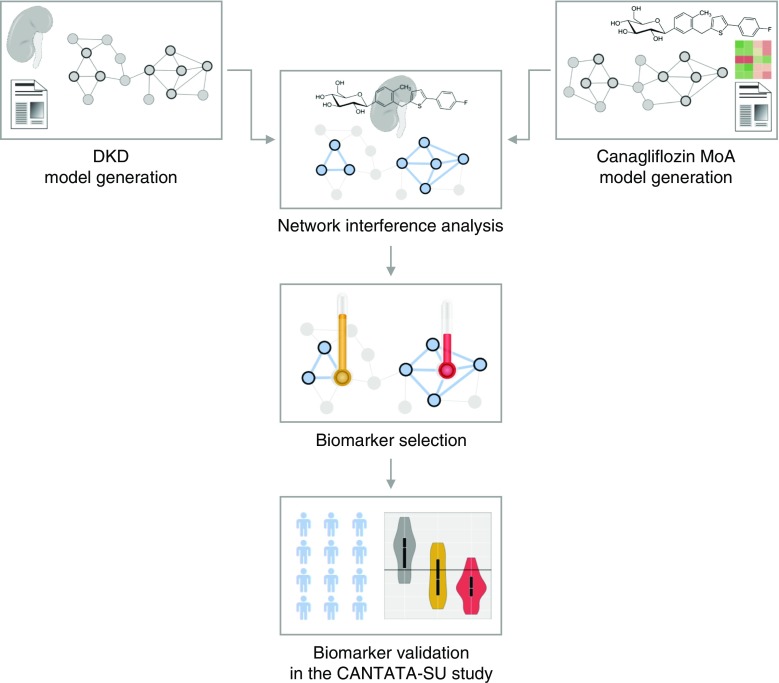
Study analysis overview scheme. Molecular models were generated based on literature-derived data as well as in vitro-derived transcriptomics profiles for DKD and canagliflozin. Network interference analysis led to identification of pathophysiological DKD processes affected by canagliflozin treatment. Biomarkers in areas of network interference were selected and validated in samples from the completed CANTATA-SU clinical trial

The overall analysis workflow from in silico modelling to biomarker validation in clinical trial samples is depicted in Fig. 1. In brief, network-based molecular models were generated using omics data and literature-extracted information for DKD as well as the effects of canagliflozin reflecting disease pathophysiology and drug MoA. Subsequently, network interference analysis of the two molecular models was performed. This analysis formed the basis for the selection of biomarkers which were subsequently measured in samples from the CANTATA-SU clinical trial (ClinicalTrial.gov registration no. NCT00968812) in order to determine the effects of canagliflozin on these biomarkers.

### Cell culture experiments and gene expression analysis

Proximal tubular human kidney cells (HK2) were purchased from American Type Culture Collection (CRL-2190; Wesel, Germany) and cultured in keratinocyte serum-free medium (KSFM) containing 10% FBS, 5 ng/ml recombinant EGF (rEGF), 0.05 mg/ml bovine pituitary extract (BPE), 100 U/ml penicillin and 100 μg/ml streptomycin. Cell culture supplies were purchased from ThermoFisher Scientific, Vienna, Austria. All cells were grown at 37°C in a humidified atmosphere with 5% CO_2_. After growth to confluency, cells were deprived of serum for 24 h. Thereafter, cells were either treated with 0.5 μmol/l canagliflozin (Biozol, Eching, Germany) or left untreated, and subsequently the canagliflozin-treated or untreated cells were stimulated with high glucose (30 mmol/l; Sigma-Aldrich, Vienna, Austria). The chosen canagliflozin concentration lay between the calculated IC_50_ (half maximal inhibitory concentration) and C_max_ (peak plasma concentration) based on prior work [13, 14]. The glucose concentration was also selected on the basis of prior studies [15, 16]. After a total of 24 h, RNA was isolated with RNeasy Mini Kit (Qiagen, Valencia, CA, USA) according to the manufacturer’s protocol. RNA yield and quality were determined using a DS-11 FX+ spectrophotometer (DeNovix, Wilmington, DE, USA).

Agilent Whole Human Genome Microarrays 4.44 v2 (Agilent Technologies, Santa Clara, CA, USA) were used for gene expression profiling with four biological replicates in the treatment as well as in the control arm. Samples were randomized and blinded before analysis. All materials for microarrays were purchased from Agilent Technologies, USA. For each sample, 200 ng total RNA was used. Cyanine 3 (Cy3)-labelled complementary RNA (cRNA) was generated with the Low Input QuickAmp Labeling Kit (5190-2305) for hybridisation to oligonucleotide microarrays (Human GE 4x44K v2, G4845A). Arrays were scanned at 5 μm using an Axon Gene Pix 4000B scanner (Molecular Devices, Sunnyvale, CA, USA) and signal intensity data were extracted using Agilent Feature Extraction software (v.9.5.3.1).

Pre-processing of transcriptomics data, including background correction, normalisation, filtering and summarisation of identical sequence probes, was done using the R package Agi4x44preprocess. The 17,985 probes remaining in the analysis set were annotated with the most up-to-date annotation file (026652_D_AA_20150612.txt) downloaded from Agilent’s array webpage (www.chem.agilent.com/cag/bsp/gene_lists.asp) on 25 August 2015. Isolated statistical significance testing provides a large set of features with minor effective fold changes, a frequent finding in in vitro settings. As this hampers the interpretation of biological relevance, we used in addition a fold change criterion (>|1.2|) to identify transcripts of eventual relevance, which were successively mapped to their respective protein-coding gene in the Ensembl Gene IDs namespace. Microarray data were deposited at the Gene Expression Omnibus (https://www.ncbi.nlm.nih.gov/geo) and are available under reference number GSE106156.

### Molecular model generation and network interference analysis

The construction of the DKD molecular model was based on a set of molecular features extracted from scientific articles with a focus on diabetic nephropathy. Publications annotated with the major MeSH term ‘diabetic nephropathies’ were extracted in January 2016 and genes/markers were derived from these research articles using the National Center for Biotechnology Information (NCBI)’s gene2pubmed mapping file downloaded on 7 April 2015 from ftp://ftp.ncbi.nlm.nih.gov/gene/DATA/. Extracted molecular features were mapped to their corresponding Ensembl Gene ID and further mapped onto a hybrid protein interaction network that consists of protein-coding genes (nodes) and protein–protein interaction data from IntAct, BioGRID and Reactome combined with computationally inferred relations and interactions between members (edges) [17]. Subsequently, the DKD gene set and the respective interactions were extracted if the molecular features (nodes) shared at least one interaction with one other member. This DKD interaction model was used for subsequent interference analysis with an in silico-generated canagliflozin MoA molecular model.

The canagliflozin MoA molecular model was constructed on the basis of extraction of molecular features from scientific literature complemented by molecular features deregulated after canagliflozin treatment from the in-house in vitro experiment on human proximal tubular cells. Molecular features extracted from scientific literature consisted of those reported to be affected by high glucose in the proximal tubule and those reported to be targeted by SGLT2 inhibition. Molecular features affected by high glucose in proximal tubule cells were extracted from a review article by Vallon [18]. Molecular features targeted by SGLT2 inhibition were extracted from publications focusing on one of the following approved or experimental SGLT2 inhibitors: canagliflozin, dapagliflozin, empagliflozin, ipragliflozin, tofogliflozin, remogliflozin, sergliflozin, ertugliflozin and T-1095. Extracted molecular features were mapped to their respective Ensembl Gene IDs and further mapped on the hybrid protein interaction network. The resulting subgraph connecting the canagliflozin-associated molecular features was extended by addition of molecular features showing deregulation after canagliflozin treatment by at least 1.2-fold as compared with untreated cells in the in vitro setting and being linked to at least one of the literature-associated molecular features also showing deregulation at the transcriptional level.

Areas in the DKD molecular model affected by canagliflozin treatment were identified by network interference analysis. In other words, shared molecular features as well as shared protein–protein interactions were identified between the DKD molecular model and the canagliflozin MoA molecular model. These areas in the network were subsequently used to identify biomarker candidates that could be changed with canagliflozin therapy and be used to monitor canagliflozin response.

### Candidate biomarker selection

Interfering nodes forming part of the DKD molecular model as well as the canagliflozin MoA molecular model were evaluated as biomarker candidates. Scientific publications linked to the respective molecular features via gene2pubmed and annotated with the major MeSH term ‘diabetic nephropathies’ and the MeSH terms ‘biological markers’ and ‘prognosis’ were retrieved. Publications were further filtered for being annotated with the MeSH terms ‘humans’ or ‘disease models, animal’ or reporting results from a clinical trial. We excluded high-throughput omics profiling studies. This set of prognostic markers for which at least one publication discussed the respective marker in the context of DKD prognosis was complemented by prognostic biomarkers as described in our previous work [11]. Next to the prognostic factors that were also part of the canagliflozin MoA molecular model, we also prioritised prognostic biomarkers located in close network proximity to areas of molecular model interference as these biomarkers are mechanistically linked to canagliflozin MoA. The prioritised biomarkers were subsequently measured in plasma samples to evaluate whether their concentration changed during treatment with canagliflozin.

### Clinical study design and population

The candidate biomarkers were subsequently assessed in plasma samples from all participants with type 2 diabetes mellitus and a urinary albumin/creatinine ratio (UACR) >1.7 mg/mmol who took part in the randomised, double-blind, active-controlled CANTATA-SU trial (NCT00968812). The design and main outcomes of this trial were previously reported [19]. This trial was originally designed to assess the effect of canagliflozin 100 mg/day, canagliflozin 300 mg/day or glimepiride titrated to 6–8 mg/day based on the maximum approved dose in the country of the investigational site. Individuals with a UACR >1.7 mg/mmol were selected to enrich the population for those at higher risk of renal function decline [19]. The CANTATA-SU trial consisted of a 2 week, single-blind, placebo run-in period, a 52 week, double-blind core treatment period and a 52 week pre-specified double-blind extension treatment period during which participants continued their originally assigned treatment. Eligible participants were between 18 and 80 years of age and had type 2 diabetes, with an HbA_1c_ between 53 mmol/mol and 80 mmol/mol (7.0% and 9.5%) while receiving metformin ≥2000 mg/day (or ≥1500 mg/day if higher doses were not tolerated). The trial was done in accordance with the Declaration of Helsinki and Good Clinical Practice guidelines. Ethics committees and institutional review boards approved the research protocol. All participants provided written informed consent before any study-specific procedure commenced.

### Biomarker measurement

UACR was measured using first morning void urine samples collected at the time of randomisation and after 52 and 104 weeks. Serum creatinine and HbA_1c_ levels were measured in fresh samples at baseline and at weeks 4, 12, 18 (HbA_1c_ only), 26, 36, 44, 52, 64, 78, 88 and 104. Serum creatinine was used to calculate eGFR with the Modification of Diet in Renal Disease equation [20]. Plasma samples for future biomarker assays were collected at the time of randomisation, at week 52 and at week 104 and stored in a central laboratory at −70°C. Plasma samples were sent to the central laboratory of the University Medical Center Groningen. Biomarkers selected through the systems biology approach were measured using a standard multiplexed Luminex technology. Luminex multiplex assays (ThermoFisher Scientific, Vienna, Austria) were used as they enabled measurement of multiple biomarker simultaneously in small amounts of plasma. The coefficient of variation, as determined in our own laboratory for the measured biomarkers, ranged between 1.8% (hepatocyte growth factor) and 8.8% (IL-6). All measurements were performed using a Luminex 200 machine (Bio-Rad, Hercules, CA, USA) with xPONENT software version 4.2 Build 1324 (www.luminexcorp.com/download/xponent-software-version/), according to the manufacturer’s instructions.

### Statistics

Differences in percentage changes in biomarker levels between randomised groups during follow-up were estimated from mixed repeated measures model (MRMM). The model included treatment, visit and treatment-by-visit interaction as factors and baseline biomarker level as a covariate. All biomarkers except fibronectin 1 (FN1) were log-transformed before entering in the MRMM to alleviate the skewness of the data. Visits were included as repeated measure units from the same individual. To allow generality for the covariance structure for the repeated measures, an unstructured variance–covariance matrix was used. In the MRMM model, all participants and all data points were included. A contrast statement was used to test the difference in the percentage biomarker change between each canagliflozin group vs glimepiride. To determine which biomarkers could be used to monitor the effect of canagliflozin on changes in kidney function during 104 weeks of follow-up, we used the week 52 values, since that was the first time point at which biomarkers were measured and effects of canagliflozin were considered to be fully present after 52 weeks. We first determined Pearson correlations between changes in biomarkers and UACR at week 52. Subsequently, biomarkers that significantly changed during canagliflozin treatment were selected to determine the association between the changes in these biomarkers from baseline to week 52 and eGFR decline during 104 weeks of follow-up. An MMRM model was used for this purpose. The model included treatment and visit as factors and biomarker change and biomarker change-by-visit interaction as covariates. The model was adjusted for baseline age, sex, systolic and diastolic BP, BMI, HbA_1c_, eGFR, albuminuria and change in albuminuria from baseline to week 52. A *p* value <0.05 (two-sided) was considered to indicate a statistically significant difference. Correlation analysis between biomarkers and UACR was performed in R version 3.4.1 (https://cran.r-project.org/bin/windows/base/) using the Pearson correlation coefficient and setting the significance value to <0.001. Other analyses were performed using SAS 9.3 for Windows (SAS Institute, Cary, NC, USA).

## Results

### Selection of biomarker candidates for monitoring and predicting canagliflozin response

**Fig. 2:**
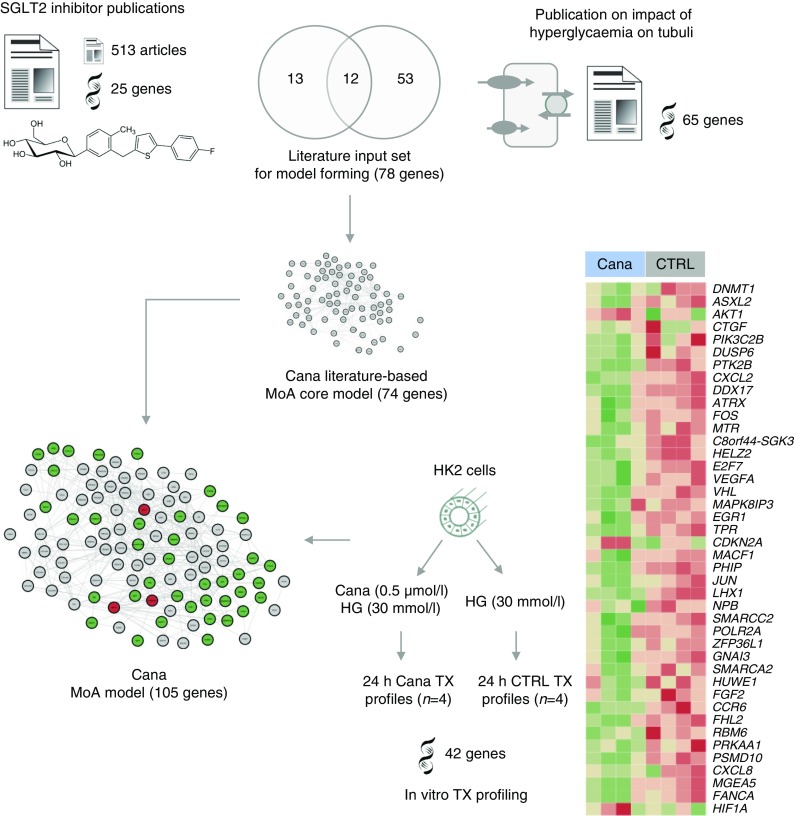
Canagliflozin MoA model construction. The literature-based MoA core model was constructed based on molecular features extracted from publications on SGLT2 inhibitors as well as from a manuscript on the impact of hyperglycaemia on tubulus cells [18]. The MoA core model (consisting of 74 connected protein-coding genes out of the set of 78) was expanded by deregulated transcripts from the in vitro cell culture experiment in HK2 cells, thus leading to the final canagliflozin MoA molecular model holding 105 protein-coding genes. Up- and downregulated protein-coding genes after canagliflozin treatment in HK2 cells are highlighted in red and green, respectively, in the final canagliflozin MoA molecular model. The intensity of colour indicates the level of up- and downregulation, with darker colours representing the greatest change. Cana, canagliflozin; CTRL, control; HG, high glucose; TX, transcriptomics. *MGEA5* is also known as *OGA*

Figure 1 shows the overall workflow. The DKD pathophysiology process model included 593 molecular features associated with DKD based on scientific literature that were interlinked at the protein–protein interaction level and thus considered for further analyses. The constructed canagliflozin MoA molecular model, on the other hand, consisted of 105 unique molecular features. The literature-based canagliflozin MoA core model consisted of 74 molecular features. Eleven of these were deregulated at the gene expression level in the in vitro gene expression study. The canagliflozin core MoA model was extended by an additional set of 31 differentially expressed genes that were linked to the deregulated genes in the core model (Fig. 2).

**Fig. 3:**
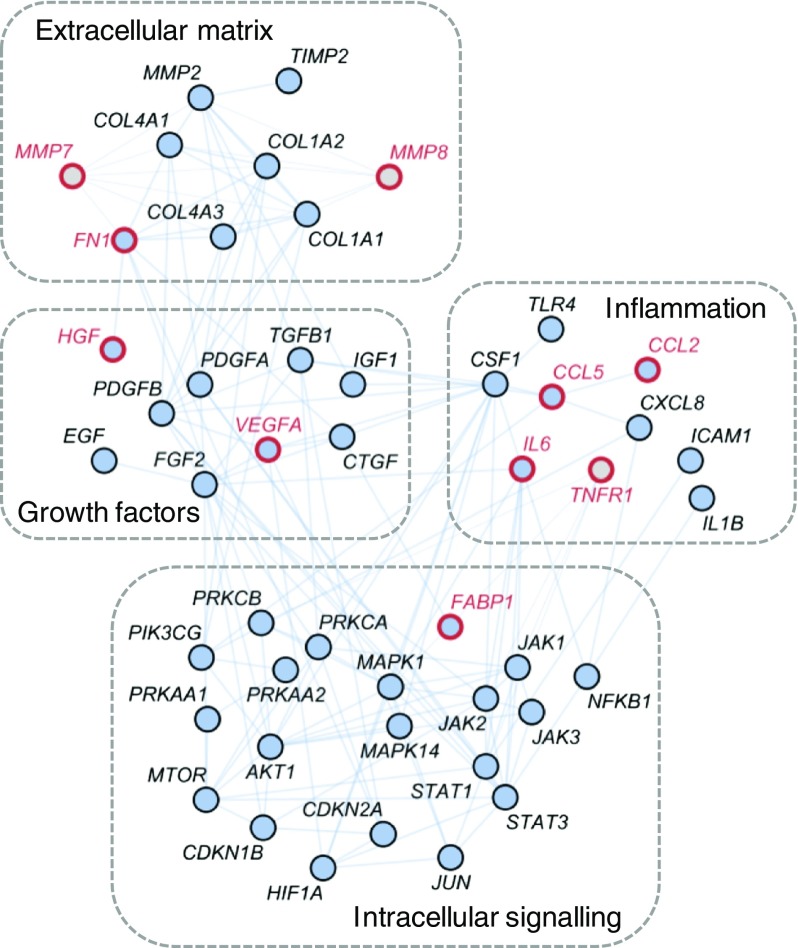
DKD–drug interference signature and selected biomarkers. The 44 protein-coding genes of the DKD–drug interference signature are shown along with genes encoding the three prognostic factors, MMP7, MMP8 and TNFR1, tightly connected to the interference signature. Protein-coding genes are grouped based on mechanistic involvement and molecular function. The ten biomarkers selected for measurements with Luminex technology in the CANTATA-SU study are highlighted in red. *CTGF* is also known as *CCN2*; *TNFR1* is also known as *TNFRSF1A*

Network alignment analysis identified 44 proteins in the DKD molecular model that were also part of the canagliflozin MoA molecular model, thus forming the network interference signature as shown in Fig. 3. These proteins were considered candidates serving as proxy for monitoring the impact of canagliflozin on pathophysiological mechanisms of DKD. For ten of the 44 proteins, literature evidence was available discussing the respective molecular feature in the context of DKD progression. The following markers were measurable with a multiplexed Luminex assay: IL-6, C-C motif chemokine ligand (CCL) 5 (also known as regulated on activation, normal T cell expressed and secreted [RANTES]), CCL2 (also known as monocyte chemoattractant protein 1 [MCP-1]), fatty acid binding protein 1 (FABP1), vascular endothelial growth factor A (VEGFA) and hepatocyte growth factor (HGF). This set was complemented by prognostic DKD markers being in close network proximity to areas of interference in the DKD and canagliflozin MoA molecular model and also measurable with Luminex technology, namely TNF receptor superfamily member 1A (TNFRSF1A), better known as TNF receptor 1 (TNFR1), as well as matrix metalloproteinase (MMP)7 and MMP8. FN1 completed the panel of selected measurable markers that were part of both generated molecular models together with evidence of them being a mechanistic marker for DKD and having prognostic potential regarding cardiovascular events.

### Biomarker changes during canagliflozin treatment

**Fig. 4:**
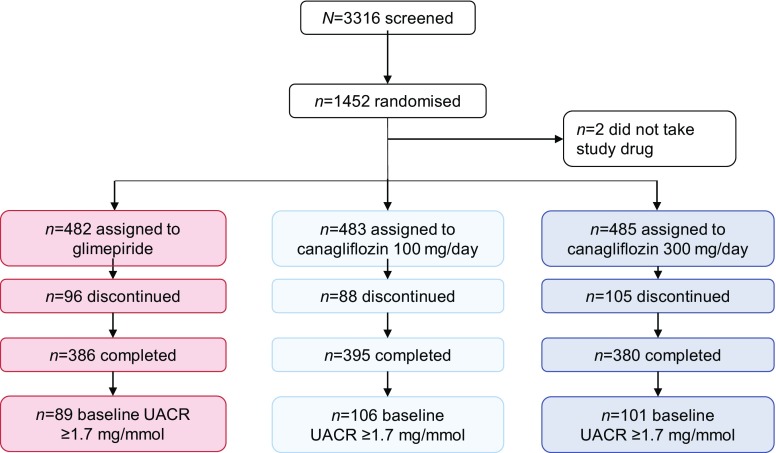
Trial profile and patient disposition of the randomized controlled trial CANTATU-SU

A total of 296 out of the 1452 participants in the CANTATA-SU trial with UACR ≥1.7 mg/mmol and stored plasma samples were selected for analysis (Fig. 4). The baseline characteristics of the population are shown in Table 1. Mean (±SD) eGFR in the overall population was 89.9 ± 18 ml min^−1^ [1.73 m]^−2^ and median (25th–75th percentile) UACR was 3.7 [2.3–7.0] mg/mmol. HbA_1c_ levels were similar at baseline and throughout follow-up among the three treatment groups. As previously presented, reductions in HbA_1c_ during follow-up among treatment groups were similar. The characteristics of the included population in comparison with the overall clinical trial population are shown in electronic supplementary materials (ESM) Table 1.

**Table 1:** Baseline characteristics of the clinical trial population

Characteristic	Glimepiride *n* = 89	Canagliflozin 100 mg *n* = 106	Canagliflozin 300 mg *n* = 101
Age, years	56.4 ± 8	56.8 ± 10	55.6 ± 9
Female sex	34 (38.2)	47 (44.3)	46 (45.5)
Systolic BP, mmHg	135.3 ± 12	134.9 ± 13	130.8 ± 14
Diastolic BP, mmHg	81.4 ± 9	80.9 ± 8	80.6 ± 7
BMI, kg/m^2^	31.7 ± 5	29.9 ± 5	30.6 ± 5
HbA_1c_, mmol/mol	63.5 ± 9	63.6 ± 9	62.1 ± 8
HbA_1c_, %	8.0 ± 0.8	8.0 ± 0.8	7.8 ± 0.8
eGFR, ml min^−1^ [1.73 m]^−2^	87.4 ± 15	89.7 ± 20	92.2 ± 18
UACR^a^, mg/mmol	4.1 (2.6–8.3)	3.8 (2.2–6.9)	3.0 (2.3–6.4)

Data are mean ± SD or *n* (%), unless otherwise indicated

^a^Median (25th–75th percentile)

**Fig. 5:**
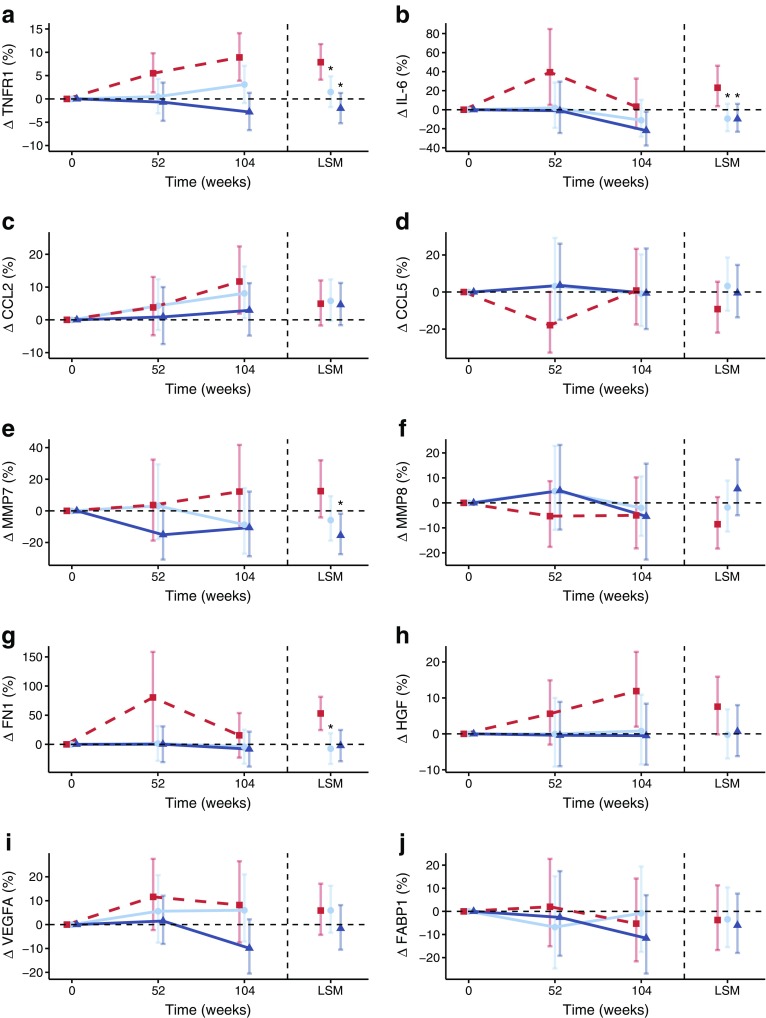
Change in biomarkers during glimepiride and canagliflozin treatment. Red squares/lines, glimepiride; light blue circles/lines, canagliflozin 100 mg/day; dark blue triangles/lines, canagliflozin 300 mg/day. Least square mean (LSM) changes are provided on each graph. Data are presented as mean ± 95% CI. *n* = 296 for all markers. *p ≤ 0.05, canagliflozin vs glimepiride

During 104 weeks of follow-up, TNFR1, IL-6, MMP7, FN1 and HGF levels increased in the glimepiride-treated group (Fig. 5). Compared with glimepiride, treatment with canagliflozin attenuated these increases or even significantly decreased the levels of these biomarkers (Fig. 5 and Table 2).

**Table 2:** Least squares mean percentage difference between canagliflozin 100 mg and 300 mg vs glimepiride

Marker	Canagliflozin 100 mg vs glimepiride	*p* value	Canagliflozin 300 mg vs glimepiride	*p* value
TNFR1	−5.9 (−10.3, 1.3)	0.013	−9.2 (−13.5, −4.7)	<0.001
IL-6	−26.3 (−41.7, −6.9)	0.011	−26.6 (−42.0, −7.2)	0.010
MMP7	−16.3 (−32.7, 4.2)	0.11	−24.9 (−39.8, −6.4)	0.011
MMP8	7.4 (−7.8, 25.1)	0.36	15.5 (−1.0, 34.8)	0.07
FN1	−15.8 (−30.9, −0.7)	0.040	−14.9 (−30.1, 0.4)	0.055
CCL2	0.8 (−7.7, 10.2)	0.85	−0.3 (−8.9, 9.1)	0.95
CCL5	13.8 (−7.3, 39.6)	0.22	9.6 (−10.9, 34.8)	0.38
HGF	−7.3 (−16.2, 2.6)	0.14	−6.4 (−15.6, 3.7)	0.20
VEGFA	0.0 (−12.7, 14.8)	0.99	−7.1 (−19.1, 6.7)	0.30
FABP1	−2.4 (−20.0, 19.1)	0.97	−2.0 (−30.8, 38.6)	0.81

Data are presented as mean (95% CI)

**Fig. 6:**
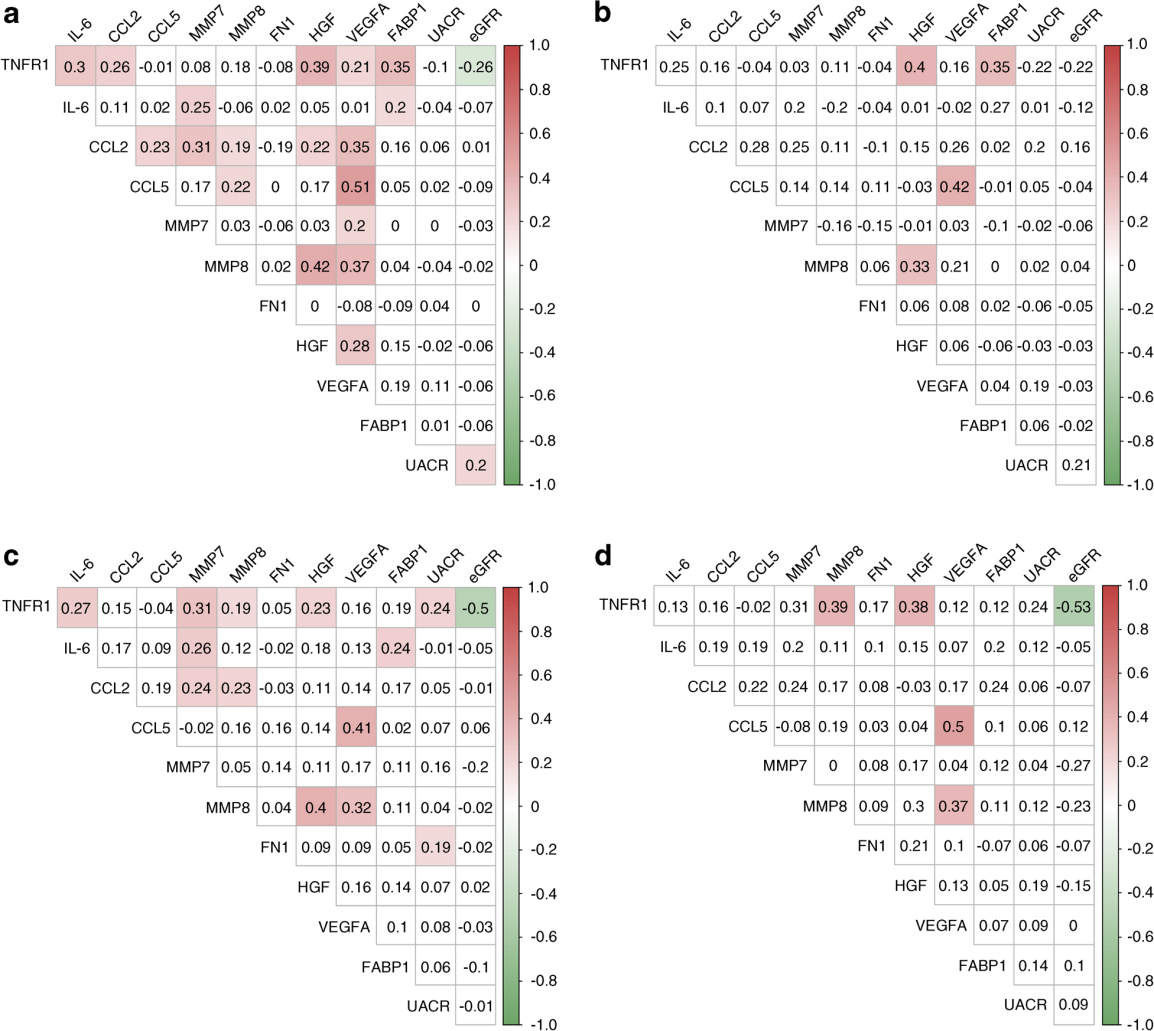
Correlation (Pearson’s *r*) between changes in albuminuria and selected biomarkers during canagliflozin treatment. (**a**) Correlation between changes in biomarkers from baseline to week 52 in the overall population. (**b**) Correlation between changes in biomarkers from baseline to week 52 in the canagliflozin 300 mg treatment group. (**c**) Correlation between achieved biomarker levels at week 52 in the overall population. (**d**) Correlation between achieved biomarker levels at week 52 in the canagliflozin 300 mg treatment group. Red shading, statistically significant positive correlations; green shading, statistically significant negative correlations

Compared with glimepiride, canagliflozin 100 mg and 300 mg decreased albuminuria over 104 weeks by 12.7% (95% CI −8.3, 29.7%; *p* = 0.214) and 35.7% (95% CI 20.1, 48.3; *p* < 0.001), respectively. Biomarker changes or achieved biomarker levels in the overall population and during canagliflozin 300 mg treatment at 52 and 104 weeks did not correlate, or only modestly correlated, with albuminuria changes or achieved albuminuria levels at both time points (Fig. 6 and ESM Fig. 1). Additionally, generally weak positive correlations were noted among biomarkers both at week 52 and week 104 (Fig. 6 and ESM Fig. 1).

**Fig. 7:**
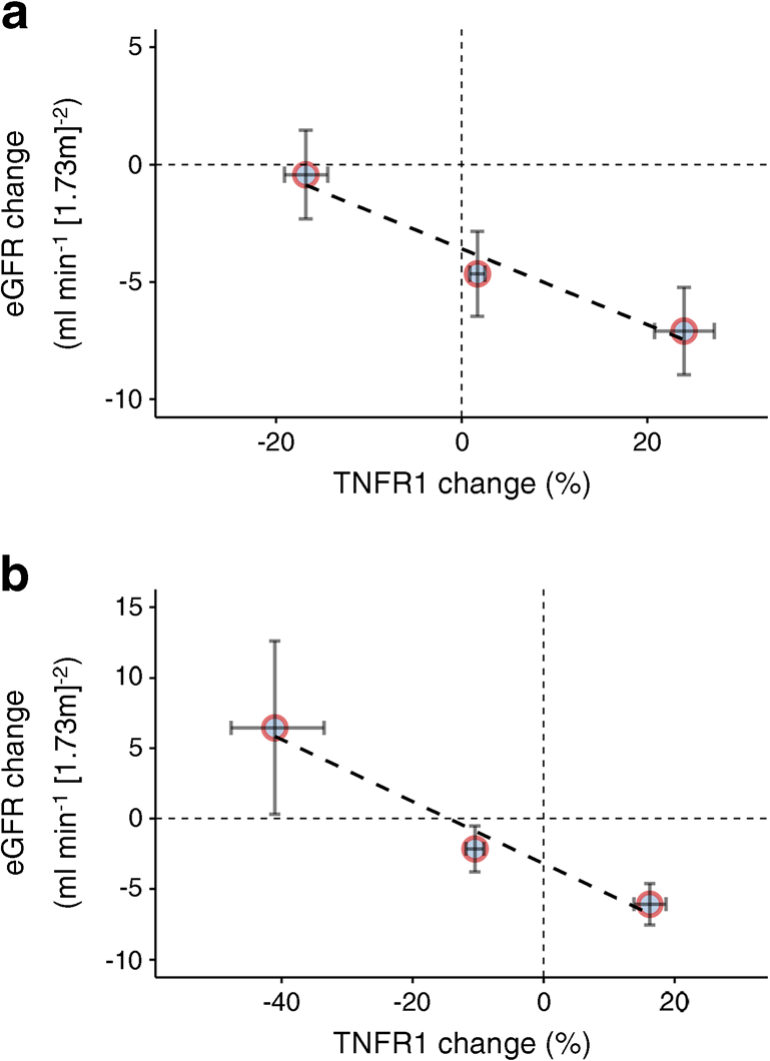
Association between change in TNFR1 and change in eGFR during follow-up. (**a**) TNFR1 change stratified in tertiles (*n* = 98 in each tertile). (**b**) TNFR1 change stratified in subgroups: ≥30% TNFR1 reduction (*n* = 9); TNFR1 reduction between 30% and 0% (*n* = 128); >0% TNFR1 increase (*n* = 157)

A further analysis evaluated whether biomarker changes can be used to monitor the treatment effect of canagliflozin. To this end, the association was determined between biomarker changes from baseline to week 52, the earliest available time point, and eGFR change over 104 weeks of follow-up. TNFR1 was the only biomarker for which the change was significantly associated with decline in eGFR (*p* = 0.026 when modelled as a continuous variable), independent of other risk markers of kidney function decline. Changes in other biomarkers were not associated with eGFR decline. When the change in TNFR1 was stratified in tertiles, least square mean eGFR change was −0.6 (95%CI −2.5, 1.3), −4.8 (95%CI −6.6, −3.0) and −7.6 (95%CI −9.5, −5.8) ml min^−1^ [1.73 m]^−2^ in increasing TNFR1 tertiles (Fig. 7a). Similar results were obtained when the population was stratified according to change in TNFR1: reduction ≥30%; between 30% and 0%; and >0% increase (Fig. 7b).

## Discussion

In this study we used a systems biology approach to select biomarkers for how the SGLT2 inhibitor canagliflozin may slow progression of DKD. Biomarkers at the interference of DKD pathophysiology and canagliflozin MoA included proteins associated with inflammatory processes, extracellular matrix deregulation and fibrosis. In a clinical trial setting, canagliflozin treatment indeed decreased TNFR1, IL-6, MMP7 and FN1 levels compared with glimepiride treatment, suggesting that canagliflozin treatment contributes to the reversal of molecular processes related to inflammation, extracellular matrix and fibrosis.

SGLT2 inhibitors appear to exert profound protective effects on kidney function in individuals with type 2 diabetes. The underlying mechanisms responsible for this beneficial effect are thought to be related to improvements in renal oxygenation and reducing intraglomerular hypertension and hyperfiltration. The present study suggests that, in addition to these effects, SGLT2 inhibition with canagliflozin exerts anti-inflammatory and antifibrotic effects and protects against extracellular matrix deregulation. These findings are in line with experimental studies demonstrating that in kidney tissues of animals treated with SGLT2 inhibitors markers of inflammation and fibrosis, including NF-κB, CCL2 and IL-6 decreased [8, 9].

The biomarkers, selected through the systems biology approach and changed during canagliflozin treatment, have been associated with progressive kidney function loss in other studies. Inflammation involving TNF-α has emerged as a key determinant of DKD progression. Binding of TNF to its receptor TNFR1 at the podocyte in the kidney stimulates cytokine production and inflammation. Bound TNFR1 can be shed by cleaving enzymes and is released in the extracellular space including the blood circulation. Various studies have consistently shown that circulating TNFR1 predicts risk of end-stage kidney disease in individuals with type 1 and type 2 diabetes [21–24]. These studies focused on a single TNFR1 measurement as predictor of risk for adverse kidney outcomes. Prior studies did not, however, evaluate whether interventions decrease high TNFR1 levels or whether the reduction in TNFR1 associates with reduced risk of adverse kidney outcomes. To our knowledge only one other drug apart from canagliflozin, namely the Janus kinase 1 (JAK1)–signal transducer and activator of transcription 2 (STAT2) inhibitor baricitinib, has been shown to decrease TNFR1 levels in individuals with type 2 diabetes [25]. However, whether reductions in TNFR1 after baricitinib treatment were associated with reduced eGFR decline could not be determined due to the short follow-up of the respective study. This study reveals for the first time that reductions in TNFR1 levels are independently associated with a lesser degree of kidney function decline. This suggests that TNFR1 is not only a strong risk marker but may even be a target for treatment.

IL-6 is another important inflammatory mediator that is targeted by canagliflozin. Triggered by hyperglycaemia, the release of IL-6 in podocytes, mesangial cells and tubule cells contributes to and sustains local and systemic subclinical inflammation [26]. Genetic studies have linked *IL6* gene polymorphisms with progression of kidney function decline, highlighting the important role of IL-6 in kidney disease [27].

MMPs are involved in collagen metabolism and are associated with fibrotic processes in the kidney. Previous studies showed that circulating levels of MMPs, including MMP7, are elevated in individuals with DKD and correlate with renal function [28, 29]. Interestingly, our in vitro tubular cell experiment showed that hypoxia-inducible factor 1α (HIF1A) was an essential feature of the DKD pathophysiology model and the canagliflozin MoA model. Intrarenal hypoxia is typically observed in diabetic kidneys and improvements in intrarenal oxygen tension during SGLT2 inhibition may in part account for the protective effects of SGLT2 inhibitors, as reviewed elsewhere [30]. Future studies using urine samples are needed to validate HIF1A as a candidate marker for monitoring response to canagliflozin treatment.

We previously developed a network-based molecular interaction model of DKD to identify biomarkers associated with kidney disease progression [11]. The model accounts for the complex interplay between the different molecular processes involved and reflects the complex pathophysiology of DKD. Model-derived biomarker panels significantly added to prediction of kidney function decline in individuals at early and advanced stages of DKD [11]. In the current study, an updated DKD molecular model was used and a network-based MoA molecular model for canagliflozin was developed using a similar bioinformatics analysis approach. Subsequent network interference analysis identified deregulated pathophysiological DKD processes potentially affected by canagliflozin treatment. This interference signature enabled an MoA-based biomarker identification for validation in a clinical trial setting. Our approach has the advantage of increasing the probability of success by incorporating pathway and process information in the selection procedure.

There are also limitations to our approach. We acknowledge that not all selected biomarkers changed during canagliflozin therapy, indicating that there is room for improvement regarding our network-based selection strategy. In the current network alignment analysis we used undirected biological networks and solely focused on the co-occurrence of protein nodes. Accounting for detailed information on network edges and weighing them either based on literature co-occurrence or co-expression in renal tissue might be an approach to focusing on critical paths within the network. The inclusion of graph measures to estimate node importance, such as degree or betweenness centrality, might add additional information on the most relevant nodes supporting marker selection. Another reason why certain selected markers did not show positive results, however, might be that measurements in the current study were restricted to plasma samples. Plasma biomarker levels are thought to reflect systemic processes and do not always specifically reflect kidney involvement. Unfortunately, urine samples from participants of the clinical trial were not used. As an example, we did not observe a reduction in CCL2 plasma levels during canagliflozin treatment in this study. It is possible that plasma CCL2 levels do not accurately reflect intrarenal CCL2 levels and that urinary CCL2 may provide a better reflection of intrarenal CCL2 levels. However, in another study of 31 patients with type 2 diabetes and high albuminuria treated with the SGLT2 inhibitor dapagliflozin, we observed a reduction in urinary CCL2 levels [31]. EGF is another interesting molecular marker reported to be associated with renal pathophysiology and progression of eGFR decline [32]. Based on molecular model interference analysis, EGF was also among the marker candidates of interest but was not selected for further analysis because EGF does not qualify for measurements in plasma samples with the Luminex technology. Connective tissue growth factor and MMP2 were also identified as promising biomarkers of canagliflozin response based on model interference but they could not be measured with the Luminex technology either. Another limitation is that we determined the effects of canagliflozin in individuals with elevated albuminuria to enrich the population at higher risk of kidney function decline. This may have affected the generalisability of the results to the broader population of individuals with type 2 diabetes who have lower levels of albuminuria. Finally, the clinical trial assessed the effect of canagliflozin against an active control, glimepiride. This has the advantage that glycaemic control among the treatment arms was similar. The effects on biomarkers are thus unlikely to be explained by the modest differential effects on glycaemic control and suggest that canagliflozin has anti-inflammatory and antifibrotic effects independent of its effect on glycaemic control. However, the lack of a placebo arm means that no definitive conclusion can be drawn on whether canagliflozin improves the biomarker levels or glimepiride worsens the biomarker levels. Thus, validation of the current set of biomarkers in larger trials in DKD, such as the Canagliflozin and Renal Endpoints in Diabetes with Established Nephropathy Clinical Evaluation (CREDENCE) trial, is required to foster the clinical implementation of these biomarkers [33].

In conclusion, this study shows that canagliflozin treatment decreases the plasma concentration of TNFR1, IL-6, MMP7 and FN1 in individuals with type 2 diabetes and elevated albuminuria. These data suggest that canagliflozin contributes to reversing molecular processes related to inflammation, extracellular matrix and fibrosis.

## Electronic supplementary material

ESM (PDF 298 kb)

## Data Availability

Data will be stored in the University Medical Center Groningen data catalogue, accessible at https://dataverse.nl. Microarray data were deposited at the Gene Expression Omnibus (https://www.ncbi.nlm.nih.gov/geo) and are available under reference number GSE106156.

## Abbreviations

CANTATA-SU: Canagliflozin Treatment and Trial Analysis-Sulfonylurea
CCL: C-C motif chemokine ligand
CKD: Chronic kidney disease
DKD: Diabetic kidney disease
FABP: Fatty acid binding protein
FN1: Fibronectin 1
HGF: Hepatocyte growth factor
HIF1A: Hypoxia-inducible factor 1α
MCP-1: Monocyte chemoattractant protein 1
MMP: Matrix metalloproteinase
MoA: Mechanism of action
SGLT2: Sodium–glucose cotransporter 2
TNFR1: TNF receptor 1
UACR: Urinary albumin/creatinine ratio
VEGFA: Vascular endothelial growth factor A
