# Supplementary material for: Canagliflozin reduces inflammation and fibrosis biomarkers: a potential mechanism of action for beneficial effects of SGLT2 inhibitors in diabetic kidney disease
Source: Diabetologia. 2019 Apr 17;62(7):1154–66. doi: 10.1007/s00125-019-4859-4 (PMC6560022; doi:10.1007/s00125-019-4859-4)

Supplementary Table 1: Baseline characteristics of patients with UACR >1.7 mg/mmol and overall clinical trial population. Data are mean (SD) or number (%), unless otherwise indicated.

	Overall population (N=1450)	UACR>1.7mg/mmol (N=296)
Age, years	56.2 (9.2)	56.3 (8.8)
Female sex, N %	694 (48%)	127 (43%)
Systolic BP, mmHg	130 (13)	134 (13)
Diastolic BP, mmHg	79 (8)	81 (8)
Body mass index, kg/m^2	30.9 (5.5)	30.7 (5.3)
HbA1c, mmol/mol	62 (9)	63 (9)
HbA1c, %	7.8 (0.8)	7.9 (0.8)
eGFR, ml/min/1.73m^2	90.2 (19)	89.9 (18)
UACR*, mg/mmol	1.0	3.7

BP = blood pressure; eGFR, estimated glomerular filtration rate; UACR, urinary albumin:creatinine ratio. *Medians are reported

Supplement Figure 1: Correlation between changes in albuminuria and selected biomarkers during canagliflozin treatment.
Panel A: Correlation between changes in biomarkers from baseline to week 104 in the overall population.
Panel B: Correlation between changes in biomarkers from baseline to week 104 in the canagliflozin 300 mg treatment group.
Panel C: Correlation between achieved biomarker levels at week 104 in the overall population.
Panel D: Correlation between achieved biomarker levels at week 104 in the canagliflozin 300 mg treatment group.
Red shaded areas indicate statistically significant positive correlations and green shaded areas statistically significant negative correlations.

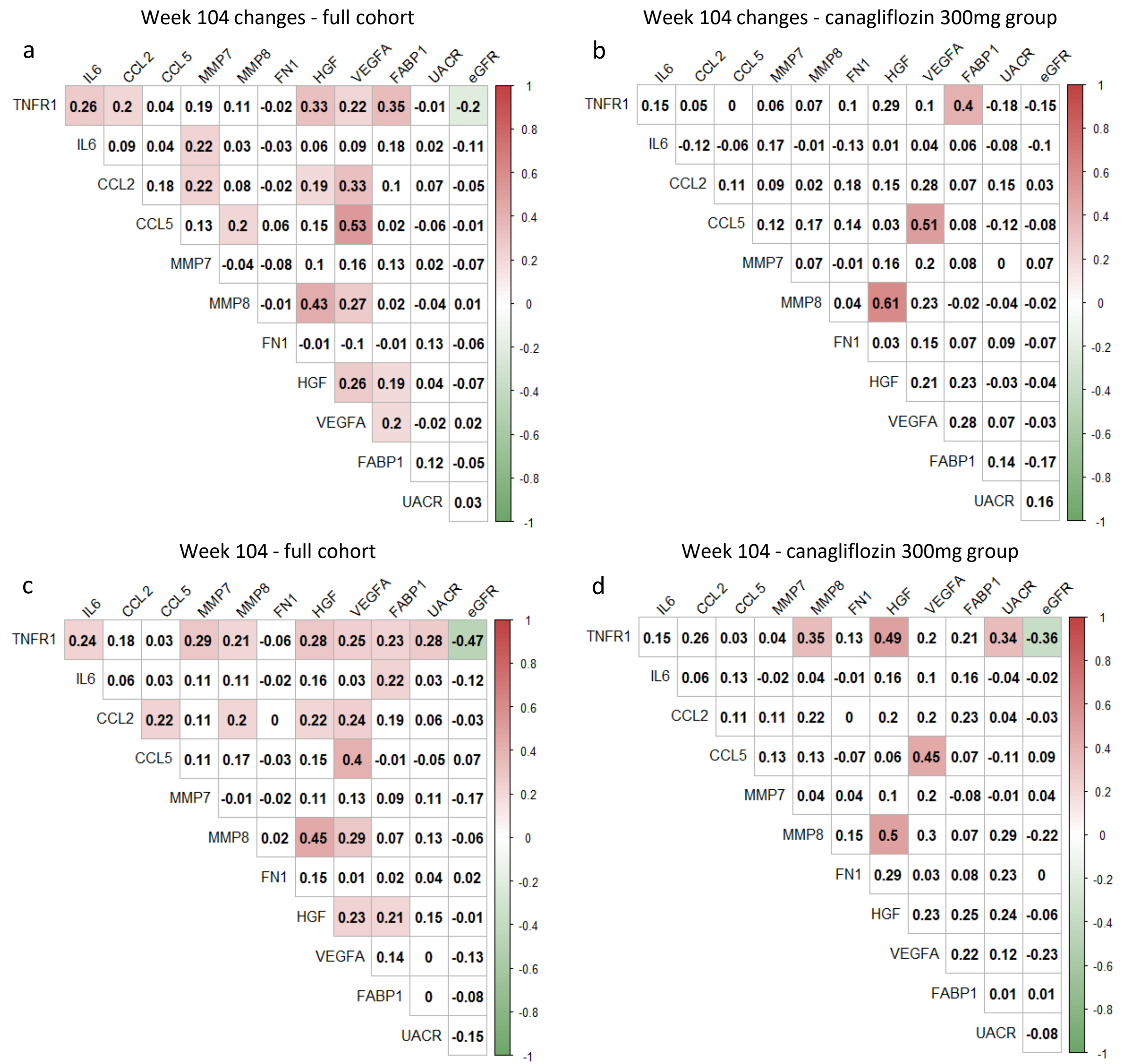

Supplement: Supplementary file 1 — (PDF 298 kb) [file 125_2019_4859_MOESM1_ESM.pdf]
